# Hirschsprung’s Disease in Adults Revealed by an Occlusive Syndrome

**DOI:** 10.7759/cureus.18484

**Published:** 2021-10-04

**Authors:** Haitam Soussan, Rachid Jabi, Mouad Ouryemchi, Zakaria Haddadi, Mohammed Bouziane

**Affiliations:** 1 General Surgery, Mohammed VI University Hospital/Faculty of Medicine and Pharmacy, Laboratory of Anatomy, Microsurgery and Surgery Experimental and Medical Simulation, Mohammed First University of Oujda, Oujda, MAR; 2 Radiology, Mohammed VI University Hospital, Oujda, MAR

**Keywords:** absence of ganglion cells, adult, hirschsprung's disease, colonic hypoganglionosis, constipation, occlusive syndrome

## Abstract

Hirschsprung's disease (HD) in adults is rare, occurring before the age of five years in 90% of cases. It is characterized by the absence of ganglion cells in a colorectal segment, resulting in functional obstruction and an upstream colonic dilatation.

HD should be considered in front of any history of chronic constipation. The diagnosis is based on a combination of clinical, manometric, radiological, and histological findings. Surgery is the basis of the treatment and consists of the resection of the aganglionic segment, followed by restoration of continuity between the two healthy segments.

We report here the case of a 20-year-old man who presented to the ER with an occlusive syndrome, which initially required a loop colostomy for decompression. History, clinical presentation, and radiological findings were suggestive of HD, but additional diagnostic methods including manometry and biopsy were employed but proved negative. Given the available data, the patient underwent a colectomy with a latero-terminal ileorectal anastomosis. Histological findings of the surgical specimen confirmed the diagnosis of HD*.*

## Introduction

Hirschsprung's disease (HD) is a malformation of the embryological development of the large intestine (colon). It is characterized by an abnormality of the motor function of the colon. This is due to the absence of the ganglion cells of Meissner's plexus in the submucosa, and those of Auerbach's plexus in the muscularis [1]. In general, the clinical manifestations of HD occur before the age of five years in 90% of cases [2]. This results in functional obstruction and an upstream colonic dilatation [3]. Surgery is the basis of the treatment.

We report here, the case of a 20-year-old man, who came to the ED for an occlusive syndrome, which required a discharge colostomy in the first stage and a total colectomy with ileorectal anastomosis in the second stage.

## Case presentation

We present the case of a 20-year-old male patient, with a history of chronic constipation. The patient presented to the ED with symptoms of large bowel obstruction consisting of abdominal pain and inability to pass gas (flatus) and stool. He denied nausea or vomiting. The patient had no history of weight loss or fever.

**Figure 1:**
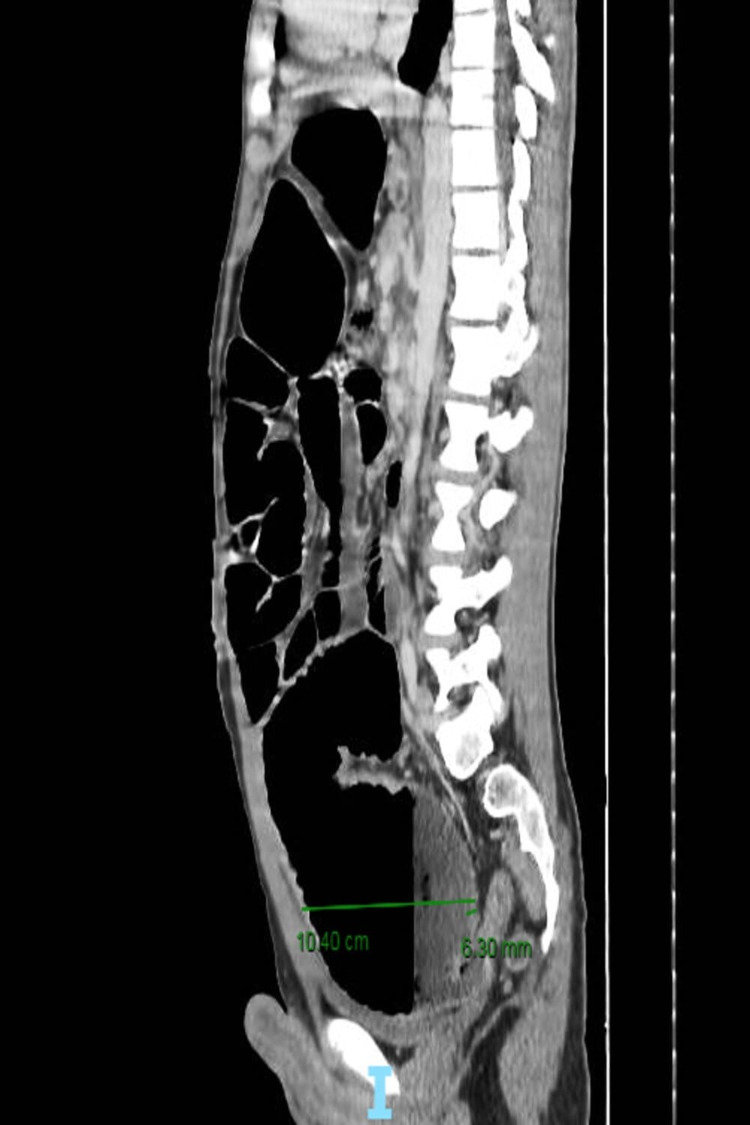
CT scan showing dilated colon and intestine in the sagittal section.

**Figure 2:**
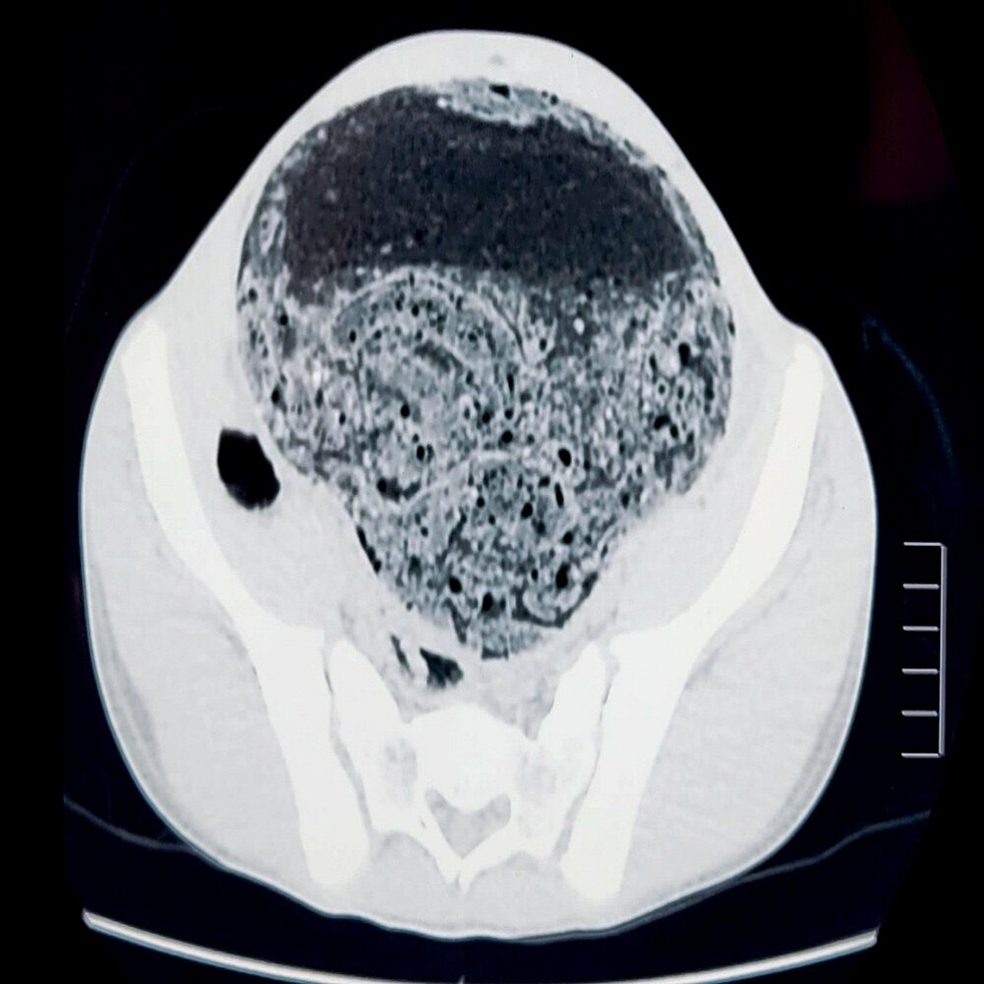
CT scan showing dilated colon in the axial section.

Physical examination revealed a conscious and hemodynamically stable patient with a distended abdomen. Palpation revealed abdominal tenderness. Digital rectal examination found a good sphincter tone, empty rectal vault. Abdominal X-ray showed multiple peripheral air-fluid levels and laboratory findings revealed leukocytosis, elevated C-reactive protein, and hypokalemia. An abdominal and pelvic CT scan was performed and showed a markedly dilated colon and intestine. The dilated colon demonstrated an abrupt but smooth transition at the sigmoid. The rectum was collapsed and appeared normal in caliber (Figures 1 and 2).

**Figure 3:**
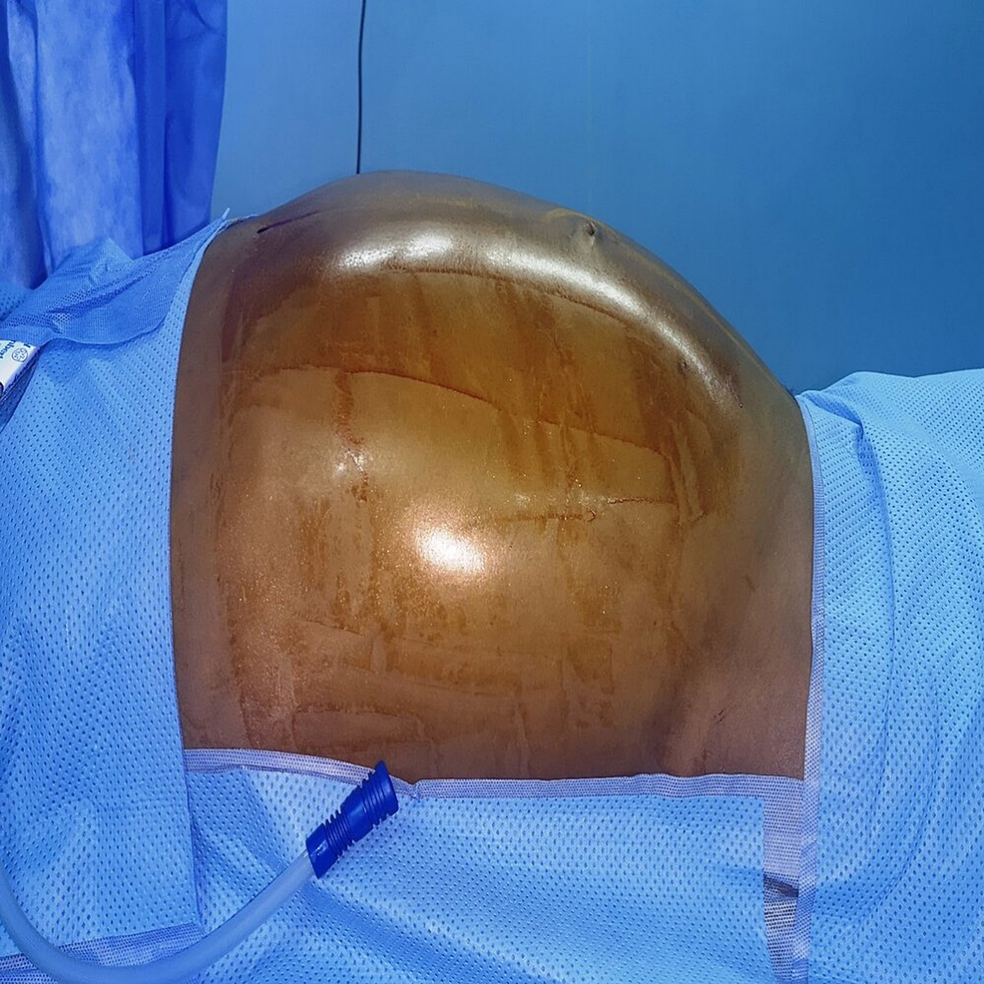
Preoperative view showing abdominal dilatation.

**Figure 4:**
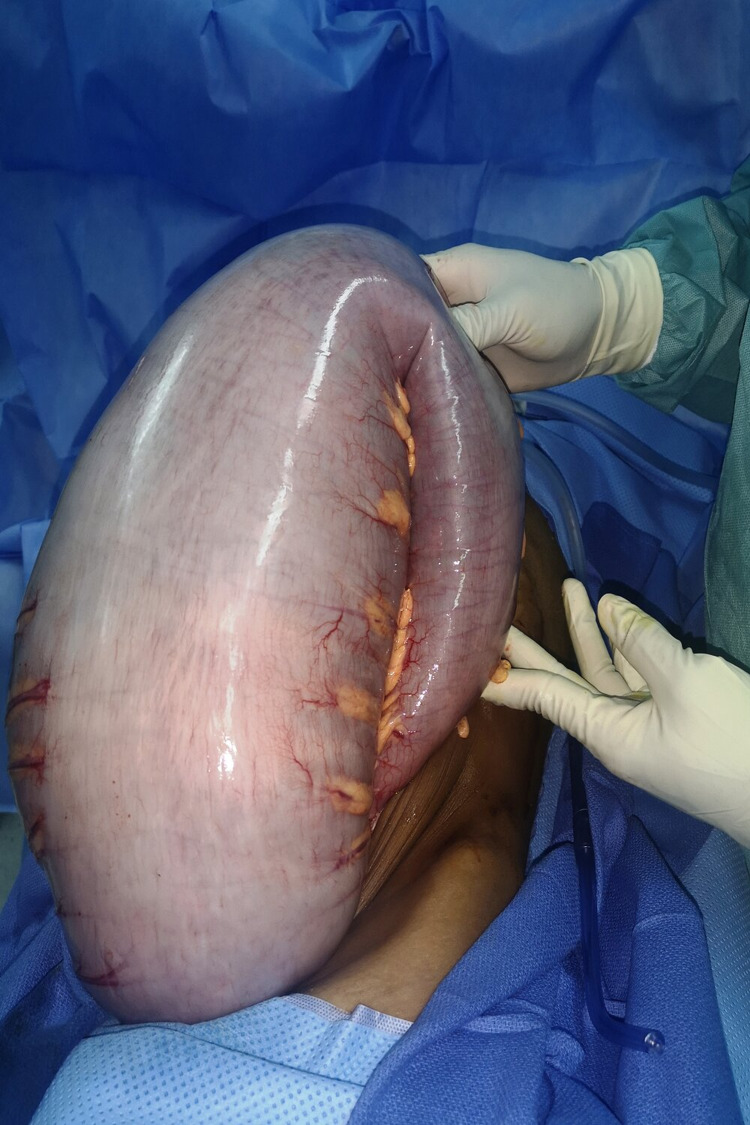
Intraoperative view showing the massive dilatation of the colonic frame.

Based on these findings, the decision was to perform a subumbilical midline laparotomy, which revealed massive dilatation of the entire colonic frame (Figures 3 and 4).

The rectal caliber was normal, with no palpable tumor. We decided to perform a left iliac colostomy. The postoperative follow-up was simple. The stoma was functional.

History, clinical presentation, radiological, and surgical findings were suspicious of HD and pushed us to practice more tests that could confirm the diagnosis. Thereafter, the patient underwent a postoperative colonoscopy, which did not reveal any abnormalities. Multiple biopsies were performed to exclude neoplastic disease and to establish the diagnosis of HD, but the histological analysis was negative. Finally, an anorectal manometry test was done and has objectified sphincter hypotonia with preserved rectoanal inhibitory reflex.

**Figure 5:**
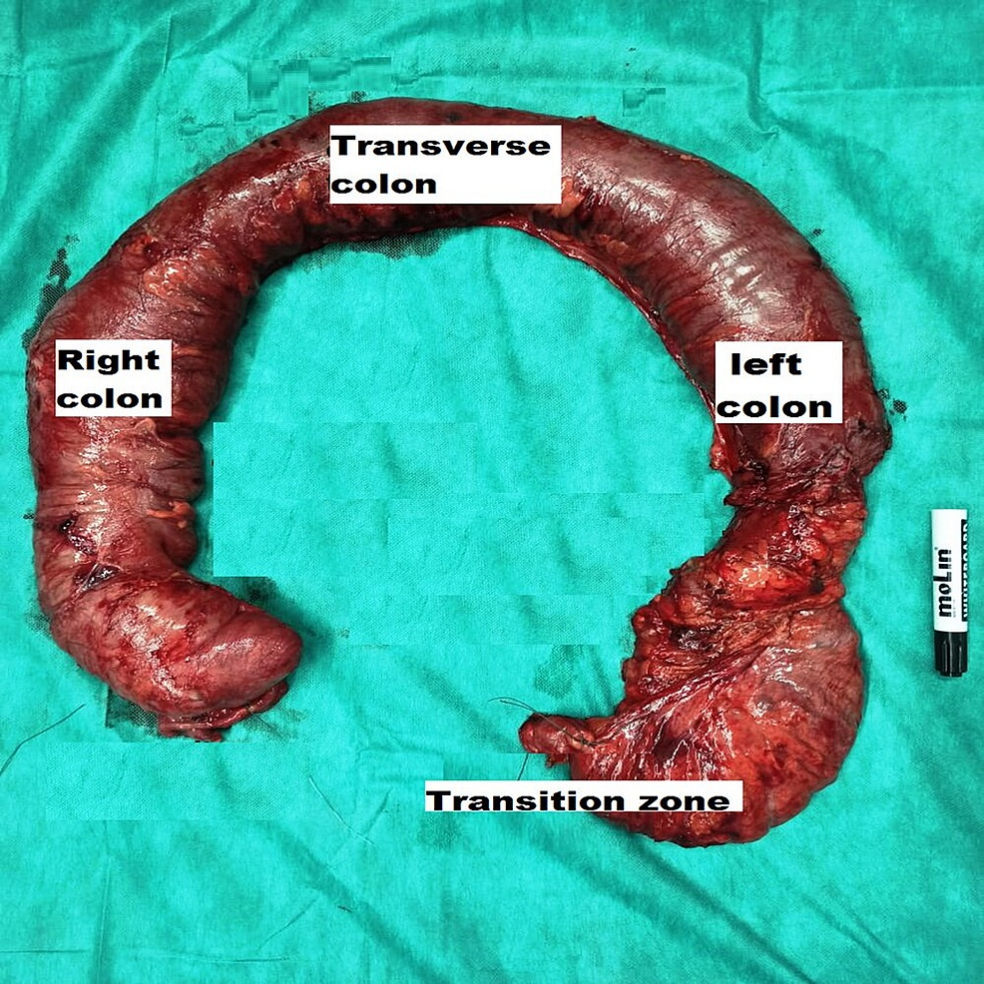
Photo of the surgical specimen showing total colectomy.

Because of the strong suspicion of HD, even with negative manometry and biopsies, and for diagnosis and therapeutic purposes, we opted for a total colectomy with a latero-terminal ileorectal anastomosis and abdominal cavity drainage, three weeks after the discharge colostomy (Figure 5).

**Figure 6:**
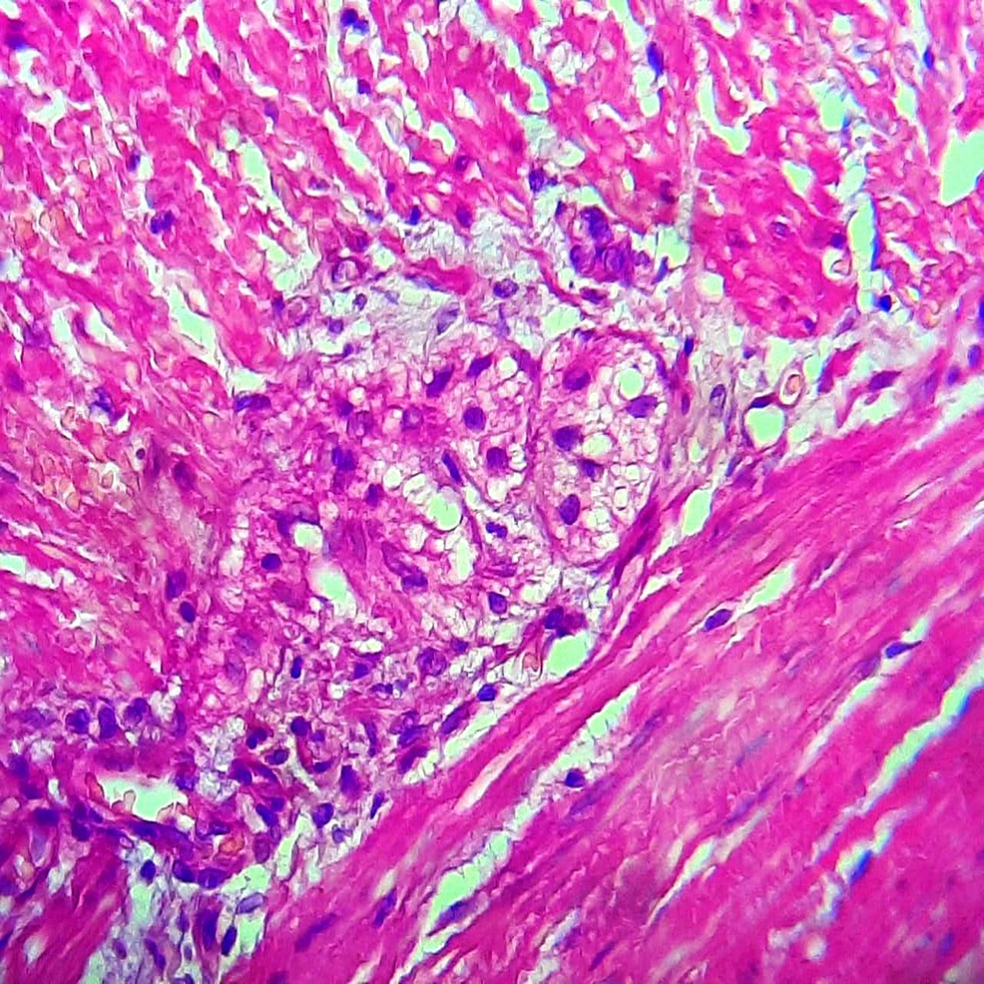
Microphotography showing Schwann cell hyperplasia and absence of ganglion cells.

Post-operative follow-up took place without incident, with an early resumption of intestinal transit. We noticed an estimated weight gain of 7 kg per year and evident improvement in quality of life. Histopathological findings showed Schwann cell hyperplasia and the absence of ganglion cells at the level of sigmoid middle part submucosa and muscularis (Figures 6 and7).

**Figure 7:**
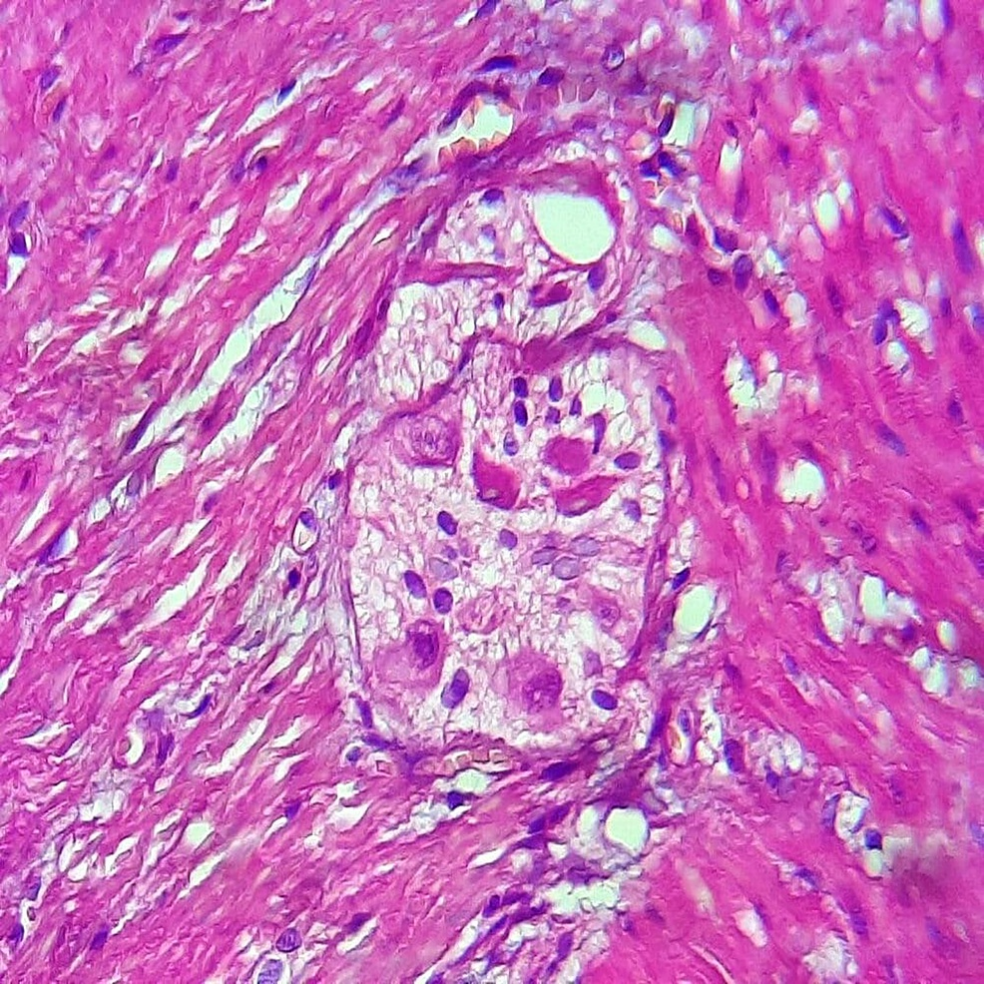
Microphotography showing the presence of ganglion cells.

Mature ganglion cells were found within Meissner's and Auerbach’s plexus in the upper half of the sigmoid, descending colon, transverse colon, and ascending colon. Given these data, the diagnosis of HD was established.

## Discussion

HD affects approximately 1 in 5000 births. Only a few are diagnosed after the age of five years [2]; of which 600 cases had been diagnosed in adulthood [3- 4]. Many authors agree that the "adult Hirschsprung's disease" diagnosis is made for 10 years old and older patients. Typically, the age of adult patients ranges from 10 to 73 years, with an increase of HD incidence before the age of 30 [3]. The most accepted theory of the cause of HD is that there is a defect in the craniocaudal migration of neuroblasts originating from the neural crest, a process that begins at four weeks of gestation and ends at week 7 with the arrival of neural crest-derived cells at the distal end of the colon. In HD, the cells fail to reach the distal colon, rendering that segment aganglionic and therefore with abnormal motor function, resulting in HD [5].

The clinical presentation differs according to age. In newborns, bilious emesis, abdominal distension, and delayed meconium emission are the main symptoms [6]. In adults, authors suggest that the delayed onset of symptoms, which are usually minimal, may be explained by the fact that the aganglionic segment is short or ultra-short [7], making it difficult to diagnose HD in adults. A history is an important tool for the diagnosis. It reveals symptoms such as abdominal pain and chronic constipation. An important part of history includes defining the nature and duration of constipation. The history should also focus on identifying secondary causes of constipation. A recent and persistent change in bowel habits, if not associated with a readily definable cause of constipation (eg, medications), should prompt an evaluation to exclude structural bowel changes or organic diseases. This is particularly important in older adults who complain of excessive straining or a sense of incomplete evacuation, or who also exhibit anemia or occult GI bleeding [8].

Noninvasive tests help to guide the diagnosis. Barium enema and CT scan show colonic dilatation, a narrowing image matching with the aganglionic zone, and eliminate other causes of chronic constipation [9-10]. Endoscopic examinations; mainly anorectal manometry shows an absence of anorectal inhibitory reflex [7-11].

As in children, in our practice at (the department of general surgery, Mohammed VI University Hospital, Oujda, Morocco), we generally perform a contrast enema and anorectal manometry rather than suction biopsy as the initial diagnostic option. If manometry findings are suggestive of HD and a clear transition zone is seen on barium enema, the study is virtually pathognomonic of HD and helps us plan the operative approach but need to be confirmed on suction biopsies findings. In case the tests described above are negative, diagnosis is based on histology findings of surgical specimens.

The diagnosis of HD is established if ganglion cells are absent in the biopsy. Supportive findings include the presence of hypertrophic nerve fibers, increased acetylcholinesterase activity or staining in the muscularis mucosae, and decreased or absent calretinin-immunoreactive fibers in the lamina propria [5].

The treatment of HD is mainly surgical and should be considered as soon as possible, given the risk of complications [10]. The objective of the treatment is the resection of the aganglionic segment, followed by restoration of continuity between the two healthy segments [5]. Thus, the three main techniques are the Swenson, Duhamel, and Soave procedures. Duhamel technique [11] consists of a retro-rectal transanal pull-through and does not require transection of the rectum [12-13]. It is generally performed in two stages [14]. The first consists of sectioning the rectum by the upper approach above the aganglionic zone, then lowering the upstream colon by a transanal approach. The second stage is based on a low colorectal anastomosis by transanal approach [10]. Swenson [15] procedure includes a leveling procedure where extra mucosal biopsies are taken along the antimesenteric border of the sigmoid to determine the level of ganglionated bowel. After the release of the pathological colon, the rectum is dissected at the contact of its wall. At this level, a stapling (lower than the Duhamel technique) then a sigmoidorectal resection and an eversion of the rectal stump are performed, followed by a colo-anal anastomosis. Soave procedure [16] consists of stripping the rectal mucosa and the muscular cuff of the rectum is maintained (similar to Duhamel's technique); a colo-anal anastomosis is performed after bringing down a ganglionic segment of the colon [13]. Swenson and Soave techniques are performed transrectally and require a temporary upstream stoma.

According to some authors, surgery can have good and satisfying outcomes by considering a less invasive technique using laparoscopic sigmoidorectal resection followed by a transanal lowering of the healthy colon without a stoma. This approach provides a better aesthetic and functional result than the techniques described above [17].

As for our patient, the therapeutic choice was difficult given the emergency context and the diagnosis doubt, as the initial investigations, including biopsies, were negative. Therefore, we went for total colectomy followed by a latero-terminal ileorectal anastomosis; histological finding of the surgical specimen confirmed HD.

## Conclusions

HD in adults is rare. It should be considered in front of any history of chronic constipation. The diagnosis is based on a combination of clinical, manometric, radiological, and histological evidence. It is defined histologically by the absence of ganglion cells in a colonic segment.

A history is an important tool for the diagnosis. An important part of history includes defining the nature and duration of constipation. The history should also focus on identifying secondary causes of constipation. A recent and persistent change in bowel habits, if not associated with a readily definable cause of constipation, should prompt an evaluation to exclude structural bowel changes or organic diseases.

The treatment of HD is mainly surgical and can be performed for diagnostic purposes. It consists of the resection of the aganglionic segment followed by restoration of continuity between the two healthy segments. The laparoscopic approach could be the basis of the treatment in the future, considering its better aesthetic and functional outcome compared to traditional procedures.
